# Natural solutions for glowing skin: spices

**DOI:** 10.3389/fnut.2025.1703354

**Published:** 2025-11-21

**Authors:** Ebru Ozler, Nevin Sanlier

**Affiliations:** Department of Nutrition and Dietetics, School of Health Sciences, Ankara Medipol University, Ankara, Turkey

**Keywords:** spices, skin, aging, anti-aging, skin health

## Abstract

Skin aging, a biological process that occurs with changes in skin appearance as a result of a decrease in physiological functions, is among the important problems of modern times for both women and men. Spices can alleviate skin aging due to their rich contents of bioactive compounds, including antioxidants and polyphenols, and can reduce the oxidative stress and inflammation that contribute to skin aging. It is thought that the antioxidant and anti-inflammatory effects of the bioactive components in spices may have positive effects on skin health with anti-aging properties. Some spices achieve protective effects against skin aging by reducing the negative effects of ultraviolet (UV) rays, proinflammatory cytokines, tyrosinase, and melanin synthesis, as well as inhibiting collagenase and elastase activity, suppressing the messenger ribonucleic acid (mRNA) expression of matrix metalloproteinases, and increasing collagen synthesis. This review addresses the promising anti-aging benefits of spices for skin health and offers some suggestions for future research.

## 1 Introduction

Aging is a biological process that occurs in response to many stress factors, causing irreversible and progressive damage to the physical functioning of all organs of the body. Genetic and environmental factors contribute to the aging process (1). The skin is the largest organ of the human body and it protects the body against some environmental factors (2). Skin consists of the dermis, composed of connective tissue, and the epidermis, composed of epithelial tissue (3). Two main sets of mechanisms are effective in skin aging. The first of these comprises internal factors, including age and genetics, and the second comprises external factors, including environmental pollution and UV rays. Aging caused by internal factors is defined as chronological or intrinsic aging, while aging caused by external factors is defined as extrinsic aging or photoaging (2, 3). With increasing age, internal and external factors cause wrinkles, dryness, epidermis thinning, and reduced barrier integrity of the skin (4, 5).

Spices have been used for many years in the kitchen for flavor, aroma, and color and for medicinal purposes in the treatment of diseases. They also exert antioxidant and anti-inflammatory effects (6).

Nutrition is of great importance for the processes that occur in skin aging (3). In addition to nutrients, it is thought that the bioactive components found in spices may have anti-aging effects together with their antioxidant effects (7). Spices such as turmeric (*Curcuma longa*) (8), ginger (*Zingiber officinale*) (9), clove (*Syzygium aromaticum*) (10), cinnamon (*Cinnamomum verum*) (11), and rosemary (*Rosmarinus officinalis*) (12) have been reported to exert positive effects on skin health and possess anti-aging properties.

Many reviews in the literature have addressed the effects of individual spices or natural polyphenols on skin aging. For example, Nie and Li (13) reported that curcumin, the active component of turmeric, may prevent skin photoaging, while Damayanti and Riyanto (14) indicated that saffron possesses photoprotective effects. Ahmed et al. (15) reviewed natural anti-aging products, and Hernandez et al. (7) discussed the anti-aging effects of nutricosmetic and cosmeceutical products. Unlike previous studies, this review comprehensively addresses multiple spices.

This review study was conducted to examine the clinical effects and mechanisms of action of spices on skin health and aging. The primary focus of this review is on the effects of oral intake. However, the number of studies on oral intake in the literature is limited. Therefore, to achieve a more comprehensive understanding of the potential mechanisms and to compare different forms of use, relevant *in vivo, in vitro*, and topical application studies were also included.

## 2 Methods

A comprehensive literature search was conducted to identify relevant studies on the relationships between spices and skin health and anti-aging effects. The literature search was conducted using the PubMed, Web of Science, Scopus, and Google Scholar databases. Articles published in English between January 2012 and January 2025 were evaluated. The following keywords were included in the literature search: “skin health,” “aging,” “skin aging,” “anti-aging,” “dietary supplement,” “nutraceuticals,” “spices,” “turmeric,” “thyme,” “hot pepper,” “black pepper,” “sumac,” “coriander,” “cumin,” “rosemary,” “mint,” “basil,” “fennel,” “saffron,” “ginger,” “clove,” “cinnamon,” “vanilla,” “star anise,” “nutmeg,” “mustard.”

English-language research articles, systematic reviews, meta-analyses, compilations, clinical human and animal studies, and cell-based research studies were considered for analysis. The titles and abstracts of the identified articles were examined and their relevance to the subject was evaluated. Studies published between the years 2012 and 2025 were analyzed and 15 key articles are presented in Tables 1 and 2. In total, 118 sources were examined.

**Table 1:** Mechanisms of action of spices on skin health and anti-aging.

**Mechanism of action**		**References**
5-lipoxygenase inhibition	Spices (especially those containing quercetin, eugenol and curcumin) can inhibit the 5-lipoxygenase enzyme involved in the biosynthesis of inflammation-mediated leukotrienes.	(104, 105)
NF-κB pathway	Spices such as turmeric and ginger may have protective properties against skin cancer by suppressing inflammation with their phytochemical content.	(106, 107)
TRPV1 channel activation	Spices such as black pepper and hot pepper may have a therapeutic effect on inflammation-mediated skin problems by activating the TRPV1 channel.	(108, 109)

NF-κB, nuclear factor kappa-B; TRPV1, transient receptor potential vanilloid 1.

**Table 2:** Some *in vivo, in vitro*, animal and human studies evaluating the effects of spices on anti-aging and skin health.

**Type of study**	**Spices**	**Sample/intervention**	**Outcomes**	**References**
Human	Turmeric (*Curcuma longa*)	*n* = 28 Turmeric, 4 weeks, 2 times a day	Sebum excretion rate ↔ Transepidermal water loss ↓	(110)
*In vivo*/human	Turmeric (*Curcuma longa*)	Turmeric extract (curcumin) Turmeric extract cream (topical application)	UV-induced gelatinase ↓ Skin photoaging ↓	(111)
*In vitro*	Black pepper (*Piper nigrum*)	B16F10 melanoma cells, piperine (44 μM)	Tyrosinase (%21.51) ↓ Melanin (%37.52) ↓ It was found to be non-damaging to the skin and demonstrated depigmentation effects.	(112)
*In vitro*	Saffron (*Crocus sativus* L.)	The effect of crocin on keratinocyte glycobiology was evaluated.	ROS ↓ NF-κB ↓ UVA-induced peroxidation and inflammation ↓ Skin aging ↓	(113)
*In vitro*	Saffron (*Crocus sativus* L.)	Safranal (volatile component found in saffron)	MMP ↓ Antielastase Antihyaluronidase Anticollagenase	(114)
Animal/*in vivo*	Ginger (*Zingiber officinale*)	Skin damage in mice with UVB, 8 weeks *Zingiber officinale Roscoe* oil (three different concentrations) was used.	Inflammation ↓ IL-1β ↓ TNF-α ↓ Skin photoaging ↓	(115)
*In vitro*	Ginger (*Zingiber officinale*)	Human dermal fibroblast cells, 6-shogaol	ROS ↓ Nrf2, SOD and CAT ↑ COL1A1 ↑ and MMP1 ↓ Collagen ↑ Cytoprotective effect Antioxidant effect Antiapoptotic effect	(116)
Animal/*in vivo*	Clove (*Syzygium aromaticum*)	UVB-exposed normal human dermal fibroblasts, hairless mice *Syzygium aromaticum* 50% ethanol extract and eugenol	MMP-1, MMP-3, AP-1 ↓ NF-κB and IL-6 ↓ NFATc1 ↓ TGF-β, NF-κB ↓ Nrf2/ARE ↑ Skin barrier function and hydration ↑ UVB-induced photoaging ↓	(117)
*In vitro*/*in vivo*	Cinnamon (*Cinnamomum cassia*)	Human skin fibroblast cells (Hs68) and mice exposed to UVA irradiation Trans-cinnamic acid (20–100 μM)	ROS, MMP AP-1, c-Fos ↓ Nrf2 nuclear translocation ↑ HO-1 and γ-GCLC expression ↑ Skin photoaging ↓	(118)

AP-1, activator protein 1; CAT, catalase; COL1A1, collagen type I alpha 1; HO-1, heme oxygenase 1; IL-1β, interleukin-1β; IL-6, interleukin-6; MMP-1, matrix metalloproteinase-1; MMP-3, matrix metalloproteinase-3; MMP, matrix metalloproteinase; NF-κB, nuclear factor kappa-B; NFATc1, nuclear factor T-cell-associated 1; Nrf2, nuclear factor erythroid 2-related factor 2; Nrf2/ARE, nuclear factor erythroid 2-related factor 2/antioxidant response element; ROS, reactive oxygen species; SOD, superoxide dismutase; TGF-β, transforming growth factor beta; TNF-α, tumor necrosis factor alpha; UV, ultraviolet; UVA, ultraviolet A; UVB, ultraviolet B; γ-GCLC, gamma-glutamyl cysteine synthetase chain C.

## 3 Skin structure and skin aging

Skin plays an important role as the primary defense mechanism against many environmental factors such as fluid loss, pathogen-related infections, physical and chemical injuries, and the UV rays of the sun (16). The skin consists of three basic layers: the epidermis, dermis, and hypodermis, respectively, from the surface inwards (17). The epidermis is a nonvascular renewable tissue consisting of keratinized stratified squamous epithelium. The basic cells of the epidermis are keratinocytes, which are found in all of its layers. There is also one melanocyte for every 36 keratinocytes in the epidermis. Melanocytes are responsible for the formation of skin color and protection against UV rays (18). The dermis, which consists largely of acellular components, provides nutrition and support to the epidermis. It also provides elasticity and durability, nourishes the skin, maintains water-salt balance, serves as a defense against foreign substances, and allows for sensation perception with touch and sensory receptors (17). The hypodermis, the third layer of skin, is located under the dermis. It maintains the thermoregulatory and mechanical properties of the skin, regulates the dermal and epidermal layers, plays a role in wound healing, regulates the cycle of hair follicles, and facilitates fibroblast and keratinocyte proliferation (19).

**Figure 1:**
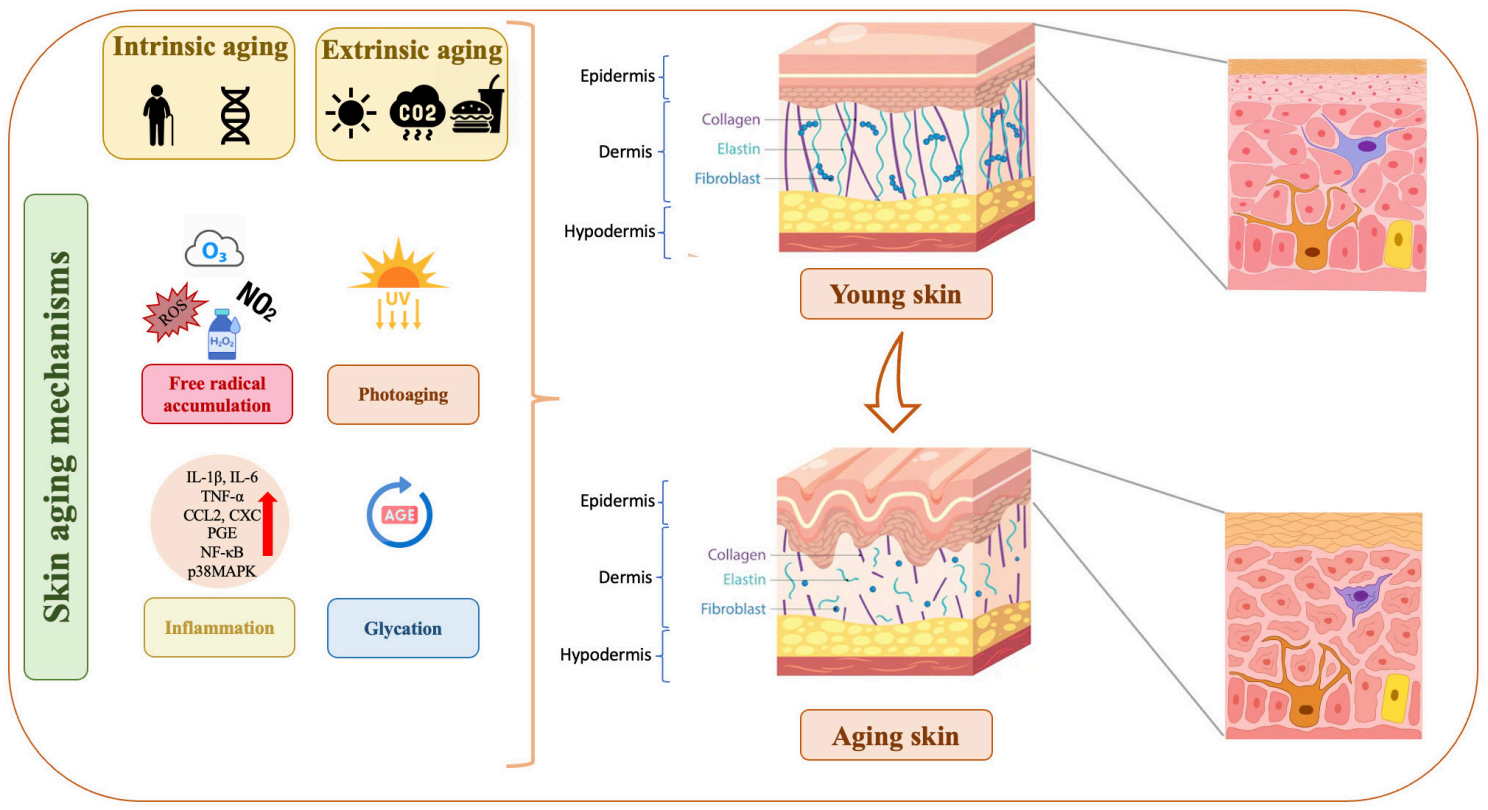
Structure of the skin and aging mechanisms.

Free radical accumulation and oxidative stress are among the factors that cause skin aging. The skin uses certain antioxidant enzymes or molecules to protect itself against reactive oxygen species (ROS). Vitamin C, vitamin E, coenzyme Q10, catalase, superoxide dismutase, and glutathione are among these antioxidants (20). Visible changes such as dark spots, wrinkles, periorbital hyperpigmentation, telangiectasia, and keratotic scales are observed with skin aging together with functional alterations including impaired skin barrier, reduced protection against mechanical stress, disrupted thermoregulation, diminished effectiveness of repair mechanisms, decreased skin elasticity, and various other biological changes (18). Sun exposure, age, gender, ethnicity, nutrition, smoking, and air pollution are among the variables that contribute to skin aging (21). The factors that cause skin aging can also be grouped as internal/intrinsic and external/extrinsic factors. Intrinsic aging is defined as aging that occurs due to genetic factors and age. As a result of intrinsic aging, dryness, pigmentation changes, loss of elasticity, and fine lines are observed on the skin. In the intrinsic aging process, epidermal renewal slows down with a decrease in epidermal stem cell reserves and keratinocyte stem cell proliferation, and skin thinning occurs (22). Aging caused by nutrition, lifestyle, and environmental factors is defined as extrinsic aging. In extrinsic aging, lentigo, coarse wrinkles, and irregular pigmentation are seen on the skin. Many cellular, molecular, and biological mechanisms including free radical accumulation, photoaging, inflammation, and glycation cause skin aging (Figure 1). The mitogen-activated protein kinase (MAPK), nuclear factor kappa-B (NF-κB), transforming growth factor beta 1/smad proteins (TGF-β1/Smad), and nuclear factor erythroid 2-related factor 2/antioxidant response element (Nrf2/ARE) signaling pathways also have effects on skin aging (7, 23, 24).

Increased levels of oxidative stress that lead to skin aging occur as a result of nitrogen oxides such as polycyclic aromatic hydrocarbons (PAH), nitric oxide (NO), nitrojen dioksit (NO_2_), and ozone (O_3_) formed by environmental pollution; increased angiotensin II and ROS production due to stress; and ROS, hydrogen peroxide (H_2_O_2_), and hydroxyl radical (•OH) caused by UV rays (25). Photoaging is an external aging process caused by sunlight and UV rays are the most important risk factor for photoaging (26). Excessive UV radiation affects signaling pathways that cause skin damage by oxidizing proteins and especially lipids and deoxyribonucleic acid (DNA) (27). Increased ROS production caused by UV radiation increases the level of tyrosinase, which is involved in melanin synthesis, and the tyrosinase-related protein 2 (TRP2) enzyme tries to reduce the ensuing oxidative stress but also increases melanin synthesis. Increased melanin synthesis causes hyperpigmentation, one of the signs of skin aging (28).

The skin's immune system is an important system in terms of the body's defense mechanisms. Mast cells, macrophages, lymphocytes, dendritic cells, and granulocytes play roles in maintaining skin homeostasis and responding to inflammation (24). UV radiation increases the production of cytokines [e.g., interleukin-1β (IL-1β), interleukin-6 (IL-6), tumor necrosis factor alpha (TNF-α)], chemokines [e.g., C-C motif chemokine ligand 2 (CCL2), c-x-c motif chemokine ligand 8 (CXCL8)], and prostaglandins. In addition, it increases inflammation by activating the NF-κB and p38 mitogen-activated protein kinase (p38MAPK) signaling pathways. All of these factors arising from UV radiation increase the inflammatory response of the skin and cause symptoms such as redness and edema (29). UV radiation stimulates cytokine production by keratinocytes in the epidermis, increases the level of TNF-α, and negatively affects the epidermal growth factor receptor (EGFR), p38 MAPK, and NF-κB signaling pathways. All of these factors cause the thinning of the epidermis and the formation of wrinkles on the skin. UV radiation damages collagen and elastin fibers in the dermis, disrupts the structure of the skin, and accelerates the skin aging process (30).

Age-related cell degeneration involves the accumulation of advanced glycation end-products (AGEs). The production of AGEs in collagen causes abnormalities in the extracellular matrix and the disruption of cell-matrix interactions. AGEs bind to receptors on immune cells and increase the release of inflammatory mediators. ROS production causes increased AGE damage. All of these factors indicate that glycation is effective in the pathophysiology of skin aging (23).

UV rays also increase the levels of matrix metalloproteinase (MMPs). Matrix metalloproteinase-1 (MMP-1) type-1 degrades collagen. MAPK pathways are regulated by proteins such as extracellular signal-regulating kinase (ERK), c-jun n-terminal kinase (JNK), and p38. UV-activated MAPKs increase the expression of activator protein 1 (AP-1), the transcription factor of MMPs (31). Furthermore, UV radiation activates NF-κB in skin keratinocytes. This activation of NF-κB leads to the expression of cytokines and inflammatory factors. It increases PGE2 in keratinocytes and also increases the expression of cyclooxygenase-2 (COX-2), which causes skin inflammation. In addition, it increases the expression of nitric oxide synthase (iNOS), which is involved in NO synthesis (7). Ultraviolet B (UVB) rays also affect the TGF-β/Smad pathway, which is the pathway for collagen synthesis. Type 1 collagen biosynthesis is stimulated by transforming growth factor beta (TGF-β). Collagen homeostasis is regulated by Smad signaling molecules. ROS production caused by UV rays reduces transforming growth factor beta receptor type II (TGF-βRII) expression, downregulates Smad3 phosphorylation, and therefore negatively affects the TGF-β signaling pathway. Increased damage to the TGF-β/Smad pathway caused by UVB radiation leads to collagen loss (31). The Nrf2/ARE pathway protects against oxidative stress. Nrf2 regulates cytoprotective proteins that have antioxidant effects, such as heme oxygenase 1 (HO-1) and glutathione synthase. In the event of oxidative stress, Nrf2 is separated from kelch-like ech-associated protein 1 (Keap1) and activated. Nrf2 migrates to the nucleus and increases the production of antioxidant enzymes. The primary function of Nrf2 in the skin is to protect against photoaging (7).

## 4 Effects of spices on skin health

Spices can positively affect both the gut microbiota and skin health. A balanced gut microbiota is associated with reduced inflammation and improved skin barrier function, and it can potentially delay skin aging (32, 33). Functional foods containing spices and other herbal products may have positive effects on health in addition to basic nutrition, reduce the risk of chronic diseases, and indirectly support skin health. The inclusion of these functional foods in the diet can improve skin appearance and achieve an anti-aging effect as a result of the bioactive compounds that they contain (34). More research is needed to identify the active compounds that are effective in suppressing the aging process and to understand their mechanisms (35). The mechanisms of action of spices on skin health and aging are shown in Table 1.

Spices have been used for centuries for their flavoring and medicinal properties and may also offer potential benefits for skin health. They contain bioactive compounds such as alkaloids, tannins, diterpenes, flavonoids, and polyphenols. Spices have antioxidant, anti-inflammatory, and anticarcinogenic properties. Their bioactive compounds, and especially phenolic compounds, have various biological effects arising from antioxidant and anti-inflammatory properties that are vital in combating skin damage and supporting skin health (6).

The bioactive component of curcumin found in turmeric (*Curcuma longa*) has antidiabetic, antibacterial, antioxidant, antiviral, antifibrotic, antifungal, and anticarcinogenic properties (36). Turmeric is used in various cosmetic products due to its skin-healing properties. It can improve skin moisture and reduce facial redness (37). In a study, healthy volunteers were exposed to UVB on the skin of the hip and received a dietary supplement containing glucoraphanin, sulforaphane (450 mg), and curcumin (1,000 mg). Curcumin and glucoraphanin were reported to be effective in reducing the expression of inflammatory cytokine genes (38). *Curcuma longa* extracts have shown antioxidant activity in relation to their ability to scavenge free radicals in DPPH and ABTS analyses. This antioxidant activity helps reduce oxidative stress, which is a major causative factor in skin aging and inflammation. In addition, *Curcuma longa* extracts exert anti-inflammatory effects by controlling NO and interleukin production in skin cells (39). Zheng et al. (8) reported that essential oil obtained from the rhizome of *Curcuma longa* reduced IL-1β and TNF-α levels, skin thickness, and cutaneous photoaging in mouse exposed to UVB light. In another study, it was determined that *Curcuma longa* had a protective effect against skin aging in a skin-on-a-chip model without a pump (40). A hot water extract of *Curcuma longa* reduced IL-1β and TNF-α and increased hyaluronan production in the skin after UVB light exposure (41). *Curcuma longa* increases skin moisture and can be used as a potential treatment for dermatological conditions due to its effect on hyaluronan production. Although turmeric is generally considered safe, excessive use or high concentrations may cause skin irritation or allergic reactions in some individuals. It is important to use high-quality turmeric products and follow the recommended dosages to avoid adverse effects. More clinical studies are needed to determine the optimal application methods and dosages for various skin problems (37).

Thyme (*Thymus vulgaris*) offers significant potential for improving skin health due to the presence of bioactive compounds such as thymol and carvacrol. These compounds possess antimicrobial, anti-inflammatory, antioxidant, and anticancerogenic properties, making thyme a promising candidate for dermatological treatments (42). *Thymus vulgaris* has anti-inflammatory properties as it suppresses proinflammatory cytokines such as TNF-α and IL-6 and enhances the levels of anti-inflammatory cytokines such as interleukin-10 (IL-10) (43). The antioxidant activities of thymol and carvacrol help neutralize free radicals and can protect the skin from oxidative stress and premature aging (42). These properties may be particular useful in formulations aiming for anti-aging effects (43). It was reported that a phytocosmetic preparation containing *Thymus vulgaris* together with lecithin increased adiponectin production, upregulated peroxisome proliferator activator receptor gamma (PPAR-γ) expression by stimulating adipogenesis, and reduced facial wrinkles and expression lines (44). In another study, it was reported that *Origanum vulgare* L., a closely related plant from the same taxonomical family, had antihyaluronidase, anticollagenase, and antielastase effects and could be protective against skin aging (45).

Hot pepper is an important spice obtained from species of the genus *Capsicum* of the family Solanaceae. Hot pepper, which is widely consumed worldwide, has rich phytochemical contents (46). The phenolic compounds found in *Capsicum annum* can protect against UV rays and exert anti-aging effects as a result of their antioxidant and anti-inflammatory properties (47). Capsaicin provides important benefits for skin health and anti-aging, has positive effects in protecting the skin from damage and aging with its antioxidant and anti-inflammatory properties, and helps reduce oxidative stress and inflammation, which contribute significantly to skin aging (48). Similarly, sweet pepper juices were found to increase the activities of antioxidant enzymes such as catalase and glutathione peroxidase and support skin health by reducing oxidative damage and preventing photoaging by regulating collagen synthesis (49). Capsaicin also reversed UV-induced collagen damage in dermal fibroblasts by reducing ROS formation, preserving skin structure and elasticity (50). Different *Capsicum* species including red pepper, Shishito pepper, and Cheongyang pepper were found to inhibit NO, ROS, and PGE2 in human dermal fibroblast cells, while Shishito pepper increased MMP-1 and procollagen I α1 levels, red pepper and Shishito pepper suppressed tumor TNF-α and showed protective properties against skin damage, and other spices obtained from *Capsicum* plants may have protective effects against skin aging (48). Thus, with these properties, *Capsicum annum* may have anti-aging effects.

Black pepper (*Piper nigrum*), the main bioactive component of which is piperine, is another key spice with antioxidant and anti-inflammatory effects (51). In a study using an emulgel formulation prepared with *Piper nigrum* extract, it was reported that the formulation inhibited tyrosinase, had an antioxidant effect and sun-protection properties, and could accordingly protect against skin aging (52). However, it is important to consider individual skin sensitivities and potential irritations caused by formulations including pepper and more research is needed.

Sumac (*Rhus coriaria*) is used for therapeutic purposes due to the antioxidant effects of the phenolic compounds that it contains (53). *Rhus coriaria* can also protect against skin aging with its antioxidant properties. It exerts protective effects against photoaging by inhibiting Ultraviolet A (UVA)-induced oxidative damage in microvascular endothelial cells (54). It also suppresses proinflammatory factors in keratinocytes via its anti-inflammatory effects and supports skin health (55). It has been reported that *Rhus coriaria* leaves have antibacterial and antifungal activities that can be effective in protecting skin health by preventing infection and supporting wound healing (56). In an *in vitro* study, it was determined that sumac extract may have protective properties against damage caused by UV rays (54). In an *in vitro* study, the effects of a gallic acid derivative phytocomplex obtained from sumac on wound healing, keratinocytes, and fibroblasts were evaluated and the phytocomplex was found to have skin-repairing properties (57). Thus, *Rhus coriaria* supports skin health and has anti-aging effects. However, more research is needed to clarify its mechanisms of action and effectiveness in human applications. In addition, the availability and cost-effectiveness of its extracts may affect its use in skin care products.

Coriander (*Coriandrum sativum*), which contains bioactive components such as sterols, terpenoids, and tocols, has antioxidant and anti-inflammatory properties (58). *Coriandrum sativum* essential oil inhibits the elastase and collagenase enzymes involved in skin aging, supports skin elasticity, and can reduce the appearance of wrinkles (59). In the study conducted by Salem et al. (59), it was further stated that coriander essential oil-loaded lipid nanoparticles and coriander oil cream reduced COX-2, prostaglandin E2 (PGE-2), MMP-1, JNK, malondialdehyde (MDA), and AP-1 levels and could reduce UV-induced skin photoaging and wrinkles. Other researchers reported that compounds isolated from coriander and fennel seeds had antiglycation effects on human fibroblast TIG-110 cells (60). Considering the role of AGEs in skin aging, these findings suggest that compounds isolated from coriander may have protective properties against skin aging.

Rosemary (*Rosmarinus officinalis*) belongs to the family Lamiaceae (61). Bioactive compounds of rosemary such as carnosol, carnosic acid, and ursolic acid have antioxidant, antimicrobial, anti-inflammatory, and wound-healing effects. With these properties, rosemary can be used in skin care and anti-aging applications to increase skin cell activity, improve skin elasticity, and reduce the appearance of wrinkles (62, 63). In volunteers exposed to UVA and UVB radiation, supplementation with rosemary and grapefruit extracts (100 or 250 mg) was found to reduce skin redness and lipid peroxides, while showing beneficial effects on skin wrinkles and elasticity (64). A topical rosemary hexane extract was found to reduce the levels of inflammatory and wrinkle markers and exert anti-aging and photoprotective effects in rats exposed to UVB light (65). In another study, it was reported that rosemary reduced ROS levels, MMP-1 and matrix metalloproteinase-3 (MMP-3) mRNA expression, and p53 protein expression and could slow down aging (12). In a study conducted by Auh and Madhavan (66), it was determined that an ethanol extract of rosemary and a hexane extract of marigold reduced inflammatory markers and could exert protective effects against skin photoaging. Rosemary essential oil also increased hair length and follicle diameter in mice exposed to UVB, prevented photoaging, and supported hair health (67). Large-scale clinical studies are needed to better understand the efficacy and safety of rosemary in dermatological applications for skin health and anti-aging effects.

Saffron (*Crocus sativus*) is a spice containing bioactive components such as carotenoids, terpenes, and zeaxanthin (68). It has therapeutic effects in cases of skin diseases with its depigmentation and skin-repairing activities (69). A study reported that saffron extract may exert skin-protective effects by inhibiting xanthine oxidase, hyaluronidase, and tyrosinase (70). Another study demonstrated that a saffron compound extract facilitated the proliferation of collagen and elastic fibers, upregulated matrix metalloproteinase-2 (MMP-2) protein, downregulated ERK1/2 protein, and could potentially reduce skin thickness and prevent wrinkle formation and photoaging (71). Habibi et al. (72) found that saffron reduced MDA and myeloperoxidase (MPO) activity, increased superoxide dismutase (SOD) activity, and increased skin flap vitality by reducing oxidative stress. Furthermore, the 12-week topical application of a cream prepared with saffron extract and avocado oil had an anti-wrinkle effect (73).

Ginger (*Zingiber officinale*) is a plant belonging to the family Zingiberaceae. It has antioxidant, anticoagulant, antitumor, and anti-influenza properties (74). Indonesian ginger species, including gajah, red, and emprite gingers, were shown to exert positive effects against skin aging, and the most effective compound in this regard was octinoxate (75). In another study, a cream containing acetyl zingerone (1%) obtained from *Zingiber officinale* was applied to the faces of healthy participants twice a day for 8 weeks and it was determined that this topical application reduced wrinkles and photodamage (9). A Japanese ginger (*Zingiber mioga*) extract (100 or 200 mg/kg) was shown to increase fibrillin-1, hyaluronan synthase 2, collagen synthesis, and elastin mRNA expression in mice exposed to UVB light for 6 weeks; it also reduced the levels of inflammatory cytokines, wrinkle-forming factors, and melanogenesis factors and improved skin moisture and depigmentation (76).

Clove (*Syzygium aromaticum*) contains bioactive components such as tannins, steroids, flavonoids, saponins, terpenoids, and alkaloids (77). It has been reported that clove reduces proinflammatory cytokines such as TNF-α, IL-1β, and IL-6; increases anti-inflammatory cytokines such as interleukin-4 (IL-4) and IL-10; downregulates NF-κB, p65, and mechanistic target of rapamycin (mTOR) mRNA expression; and protects the skin against UVB damage by regulating skin sarcomembrane Na+-K+-ATPase (78). In another study, it was stated that n-hexane and ethanol fractions obtained from clove buds and leaves induced mitochondrial activity, delayed the G1 phase of the cell cycle, and possessed antioxidant and anti-aging activities (79). Hwang et al. (10) found that clove inhibited MMP-1, MMP-2, MMP-3, and matrix metalloproteinase-9 (MMP-9) gene expression and elastase and could have antioxidant and anti-wrinkle effects.

Mustard (*Brassica juncea*) is a plant with high vitamin, mineral, fiber, and phytochemical contents (80). A nanoemulsion gel containing flaxseed oil and black mustard oil, with high omega-3 fatty acid contents, was found to exert a positive effect against D-galactose-induced skin aging, although the flaxseed oil was more effective than the black mustard seed oil (81). Fares and Radaydeh (82) stated that the combination of mustard oil and aloe vera oil may have anti-aging potential due to the antioxidant and antimicrobial effects of these plants.

Cumin (*Cuminum cyminum*) is a spice belonging to the family Apiaceae with antioxidant, anti-inflammatory, antimicrobial, anticancer, and antidiabetic properties (83). Cumin protects skin cells from damage by inhibiting free radicals with its antioxidant properties and can support a youthful skin appearance (84, 85). Cumin protects skin elasticity with the essential nutrients it contains, such as iron. It is thought that the volatile oils of cuminaldehyde and thymol in cumin support skin health by reducing inflammation (86). In a study of B16F10 murine melanoma cells, it was determined that cumin extract suppressed tyrosinase, monophenolase, diphenolase, and melanin production while reducing free radicals; therefore, it may be effective in skin whitening (87).

Peppermint (*Mentha*) contains active components such as phenolic acid, terpenoids, steroids, and flavonoids (88). A leaf extract obtained from *Mentha piperita* inhibited adenosine triphosphate (ATP) release from epidermal keratinocytes and could reduce dermal thinning and wrinkle formation (89). Apple mint (*Mentha suaveolens*) exerts antithermal activity against skin aging by inhibiting ROS and MMPs (90). Various *Mentha* species can show anti-aging effects. Thus, plants of the genus *Mentha* can be used for cosmetic and medicinal purposes (91). For example, a *Mentha piperita* leaf extract was found to reduce extracellular adenosine triphosphate (eATP) release from epidermal keratinocytes and could have positive effects against skin aging and wrinkles (89).

Basil (*Ocimum basilicum*) belongs to the family Lamiaceae and has high contents of polyphenols and phenolic acids. Researchers reported that a product containing basil extract had an anti-aging effect (92). Another study demonstrated that a basil extract containing rosmarinic acid reduced ROS and carbonylated proteins in fibroblasts after exposure to UVA light and could display protective properties against photoaging (93).

Vanilla (*Vanilla*) is a plant genus belonging to the orchid family (94). In a study examining the effect of *Vanilla pompona* on skin aging, it was reported that compounds obtained from this plant supported the synthesis of hyaluronic acid, elastin, and collagen in a normal skin cell model while significantly reducing the aging phenotype in a senescence model, and such compounds could offer important protection in the proposed UV-induced photo-senescence model (95).

Cinnamon (*Cinnamomum verum*, previously known as *Cinnamomum zeylanicum*), of the family Lauraceae, contains components including iron, fiber, manganese, polyphenol, cinnamate, cinnamaldehyde, and cinnamic acid that are widely used in nutrition and medicine (96). It was reported that cinnamaldehyde, a particularly important component of cinnamon, reduced ROS and DNA damage in human keratinocytes exposed to UVB radiation, and in a mouse model, topical application reduced dermal inflammatory cell infiltration, wrinkle formation, epidermal hyperplasia, and the negative effects of UVB radiation on collagen synthesis (11).

Nutmeg (*Myristica fragrans*) belongs to the family Myristicaceae (97). Researchers reported that nutmeg could suppress the MAPK phosphorylation induced by ROS, reduce MMP-1 expression caused by UV radiation, and exert antiphotoaging effects (98).

Star anise (*Illicium verum*) is the star-shaped fruit of a medium-sized evergreen tree and is known for its antiviral properties (99). However, star anise is also used in anti-aging cosmetic products due to its antioxidant effects (100).

Fennel (*Foeniculum vulgare*) belongs to the family Apiaceae (101). One study reported that an extract of *Foeniculum vulgare* inhibited UV-induced melanogenesis (102).

In addition to their positive effects, spices may also cause allergic reactions. Some types of spices can cause T-cell-based inflammation reactions, leading to skin problems such as dermatitis (103). Overall, however, spices have positive effects on skin health with their antioxidant and anti-inflammatory properties and various mechanisms of action such as the inhibition of 5-lipoxygenase, suppression of NF-κB, and modulation of eicosanoid production. In the literature, spices such as turmeric and rosemary have been investigated more extensively through human studies; in contrast, most studies on spices like sumac, ginger, saffron, cumin, and clove have been conducted at the *in vivo, in vitro*, or animal model level. There are also studies in the literature that include topical applications involving mustard, coriander, black pepper, ginger, and saffron. However, there are far fewer studies on the effects of basil, vanilla, fennel, star anise, and nutmeg on skin health and anti-aging compared to other spices. Although the potential of spices in skin health is promising, more research is needed to fully understand these mechanisms and their effectiveness in human applications.

Some *in vivo, in vitro*, animal, and human studies evaluating the effects of spices on aging and skin health are summarized in Table 2.

## 5 Limitations and strengths

The strength of this review is that it addresses the anti-aging and skin-protective effects of spices. A comprehensive literature review was conducted on the effects of spices on skin health and aging, and the potential mechanisms of action of different components were examined in detail. This study contributes to the literature by bringing together existing information on the subject with a multifaceted approach. The most important limitation of this review is that most of the analyzed studies involved *in vivo, in vitro*, and topical applications. Human studies to date involving oral intake of these products are quite limited, which negatively affects the generalizability of the data. More studies are needed on this subject in the future.

## 6 Conclusion and recommendations

The antioxidant and anti-inflammatory effects of the bioactive components found in spices have positive effects on skin health and possess anti-aging properties. Spices may exert protective effects against skin aging through multiple mechanisms. They can reduce the increase in ROS, TNF-α, IL-6, and IL-1β levels caused by UV exposure in the skin. They may also decrease tyrosinase activity and melanin synthesis, which can lead to hyperpigmentation. Furthermore, spices can enhance collagen synthesis, suppress MMP mRNA expression, inhibit collagenase and elastase activity, and increase the activity of antioxidant enzymes such as SOD. Although the benefits of spices in protecting against skin aging are promising, it is necessary to take into account the variability in individual responses to these compounds. Factors such as genetic predisposition, lifestyle, and environmental exposure can influence the effectiveness of spices on skin health. In addition, more research is needed to fully understand the mechanisms of spices in anti-aging formulas and optimize their use. The careful integration of spices into both diet and skin care routines offers a natural and holistic approach to preserving youthful skin. Although the potential of spices for anti-aging applications and skin health is promising, generalizations cannot be made due to the limited number of human studies in this area. More *in vivo, in vitro*, cell, animal, and human studies are needed in clinical settings.

## 7 Future perspective

In the light of the studies reviewed here, it can be stated that some spices have positive effects on skin health and aging. However, the majority of the studies in the literature to date have involved *in vivo, in vitro*, and topical applications. Therefore, the effects of spices when consumed orally are not yet clear. It is necessary to strengthen the existing evidence on this subject and collect new data.

Future research should primarily focus on the following areas: (1) conducting clinical studies evaluating the effects of oral spice intake on skin health and aging; (2) To investigate the long-term effects of dietary supplement and nutraceutical forms of spices on skin hydration, elasticity, and wrinkle formation; (3) elucidating the molecular mechanisms and bioavailability of bioactive compounds found in spices within skin tissues; (4) investigating the potential synergistic effects of spices with cosmetic ingredients and other dietary components, and developing new delivery systems to enhance their bioavailability.

This review study offers guidance for future *in vivo, in vitro*, animal, and human studies investigating the pharmacological and clinical effects and modes of action of spices on skin health and aging with the aim of supporting new generalizations on this subject.
