# Supplementary figures and images for: Natural solutions for glowing skin: spices
Source: Front Nutr. 2025 Nov 21;12:1703354. doi: 10.3389/fnut.2025.1703354 (PMC12678094; doi:10.3389/fnut.2025.1703354)

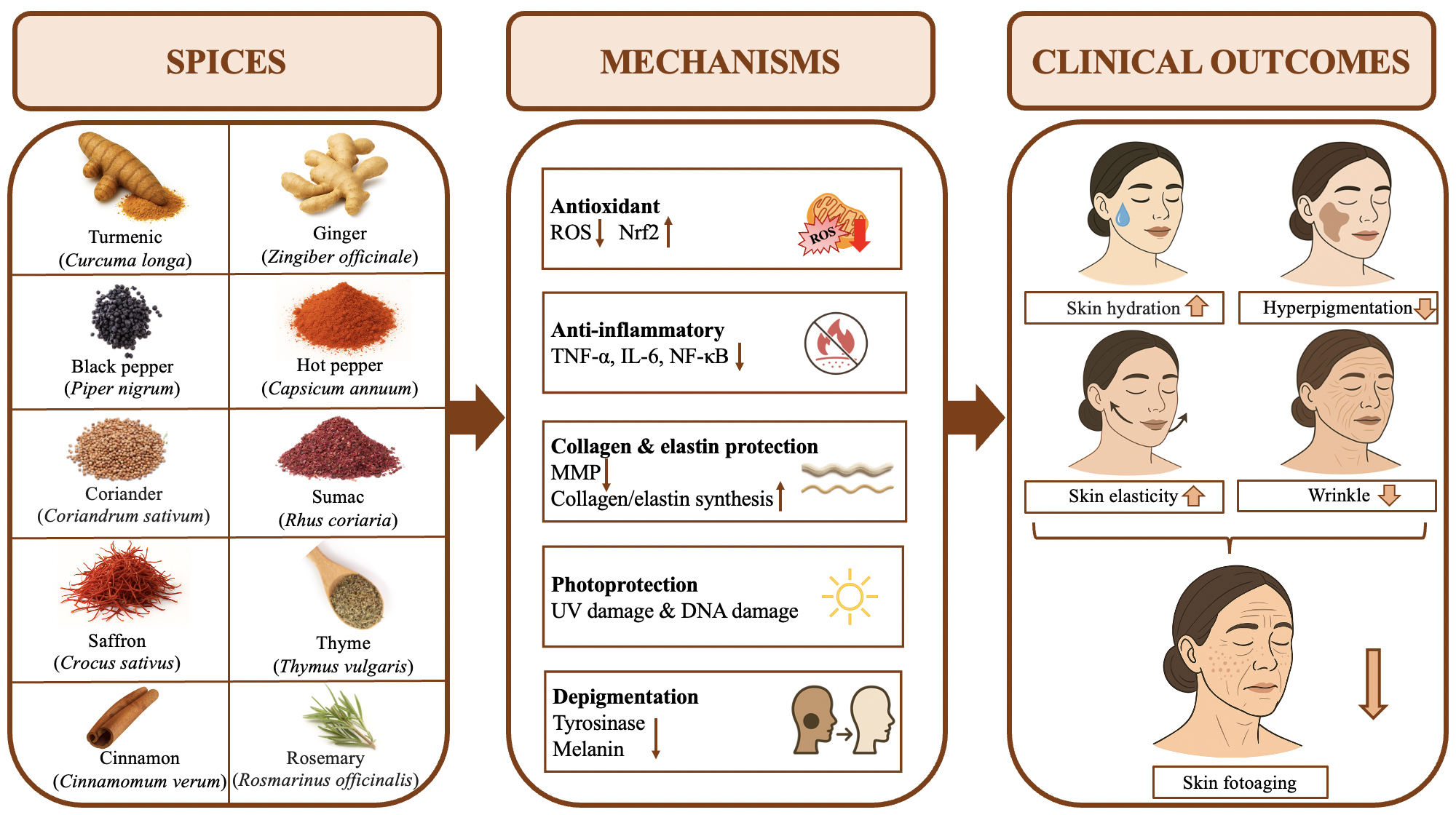

Supplement: Supplementary file 1 [file Image_1.PNG]
